# The Perspective of Brazilian Women Orthopaedic Surgeons on Gender Discrimination: Initial Insights to Understand Gender Bias in the Brazilian Healthcare System

**DOI:** 10.7759/cureus.61325

**Published:** 2024-05-29

**Authors:** Amanda Amaral, Isabela Calcado, Amparo Gomez, Carla Ricci, Verena Oberlohr, Madeline C Mackechnie, Theodore Miclau III, Vincenzo Giordano

**Affiliations:** 1 Prof. Nova Monteiro Service of Orthopedics and Traumatology, Hospital Municipal Miguel Couto, Rio de Janeiro, BRA; 2 Orthopedics Service, Hospital Universitario de la Samaritana, Bogota, COL; 3 Research Office, Arbeitsgemeinschaft für Osteosynthesefragen (AO) Foundation, Curitiba, BRA; 4 Department of Orthopaedics, University of San Francisco, San Francisco, USA; 5 Department of Orthopaedic Surgery, University of San Francisco, San Francisco, USA; 6 Prof. Nova Monteiro Service of Orthopedics and Traumatology, Hospital Municipal Miguel Couto (hmmc), Rio de Janeiro, BRA

**Keywords:** professional development, orthopaedic surgery, burnout, gender diversity, gender discrimination

## Abstract

Despite the societal progress made in recent years, gender discrimination is still common in healthcare, especially in some surgical specialties such as orthopaedics. In Brazil, where the participation of women in the medical profession has been increasing, little is known about women's perceptions on the issue of gender discrimination. This study aims to examine women orthopaedic surgeons' experiences in dealing with conflict in the workplace and contextualize the impact that gender discrimination has had or currently has on their careers and well-being. As a secondary objective, the work seeks to understand whether there are differences in the perception of the issue among practicing women orthopaedic surgeons and those in training. For a cross-sectional qualitative study, a survey was distributed exclusively to 300 practicing orthopaedic surgeons and orthopaedists in training (residents and fellows). A total of 99 women participated in the survey, of whom 66 were practicing orthopaedic surgeons and 33 were orthopaedists in training. The study showed that women orthopaedic surgeons in training in Brazil have a lower number of publications and a moderate level of involvement in academic society activity. In addition, orthopaedic surgeons in training experience a statistically significantly higher number of conflicts in the workplace. The comments from the questionnaires highlighted the physical and psychological consequences arising from these situations of professional conflict, most frequently occurring with orthopaedic surgeons who are men. Our findings indicate that respondents expressed a feeling of inequality towards women in the workplace, ultimately reducing the level of job satisfaction among female orthopaedic surgeons, which may contribute to disinterest and abandonment of the specialty. The results of this work support recent evidence that there is an implicit and often overlooked bias against the participation of women and ethnic minorities in the orthopaedic community in Brazil.

## Introduction

Despite the progress made in recent years in society at large, gender and ethnic discrimination are still common in healthcare [1]. Gender discrimination refers to any practice or belief that favours people of one gender over another; in medicine, this bias has traditionally favoured men over women, placing them in superior positions personally and professionally [2]. Addressing and preventing all forms of discrimination not only encourages inclusion but also creates a healthier environment for diversity, equity, and inclusion.

While educational initiatives aimed at gender parity are gradually being expanded, prejudice and discrimination still persist in various sectors of medical practice [2]. Despite numerous studies showing that women physicians exhibit greater empathy and sensitivity, provide more comprehensive information, and opt for simpler and more collaborative decision-making in patient care, women still tend to receive lower remuneration than their colleagues who are men [1,3-5]. Moreover, when surgical treatment is indicated, it has been shown that most patients feel more comfortable with surgeons who are men, even though it is well-known in medical circles that there is no reason to expect differences in the results of procedures performed by specialists who are men or women [6,7].

The erroneous impression that surgeons who are men are superior to their colleagues who are women in various specialties dominated by men, such as orthopaedics, creates barriers and stereotypes, which are often veiled and insufficiently discussed [1,8,9]. In a study with 373 women orthopaedic surgeons conducted in the United States of America, 72% reported having experienced some form of workplace conflict related to their being a woman, 8% reported having left or changed their workplace due to gender conflicts, and 17% said they would not choose the same specialty again if they had a second chance [8].

In Brazil, where the participation of women in the medical profession has been increasing, with more physicians who are women than men in the age group of 29 and below [10], little or nothing has been done to understand women's perceptions of gender discrimination. Only by properly knowing the problem and the existing disparities, which likely vary across medical specialties, can corrective actions be taken to achieve a balanced gender representation in the medical profession. This perception-based study aimed to examine women orthopaedic surgeons' experiences in dealing with conflict in the workplace, contextualizing the impact that gender discrimination had or has on their careers and well-being. As a secondary objective, we sought to understand whether there are differences in the perception of the problem among practicing women orthopaedic surgeons and those in training. The data collected in this study will be useful to raise awareness and develop a training model on gender discrimination in Brazil.

## Materials and methods

Following the model used in the study by Dossett et al. [11] and specifically adapted by Rodarte et al. [9] for the area of orthopaedics, a cross-sectional qualitative study was performed using a 47-question survey translated, but not validated, into Brazilian Portuguese. The terms women and men were selectively used rather than female and male throughout the investigation to delineate gender identity rather than birth sex. Questions were designed to inquire about age, orthopaedic subspecialty, years in practice, training, career path, and various scenarios of conflict in the workplace. The survey also inquired whether the respondents had encountered any complaints or reports generated from colleagues who were men or patients and, if so, what were the emotional impacts of such events. Finally, the survey featured questions about the participants' perceptions of the so-called ethnic minorities (Afro-descendants, indigenous people, and immigrants) [12] in relation to Caucasian professionals. A psychometric response scale (5-point Likert scale) was employed, in which respondents specified their level of agreement from 'fully agree' to 'fully disagree' [13].

The research was strictly anonymous and focused on scientifically exploring gender-related topics through the examination of everyday practice without collecting personal and/or health data from the participants. The study was exempt from ethical analysis, as provided by Article 1 of CNS Resolution No. 510 from 2016 [14]. All respondents signed the informed consent form included in the questionnaire.

The questionnaire was distributed to 300 women in two different ways: (i) in person at the 54th Brazilian Congress of Orthopaedics and Traumatology, held in the city of Florianópolis (Santa Catarina, Brazil), in November of 2022; and (ii) through Google Forms via social networks WhatsApp and Instagram. The president of the Brazilian Society of Orthopaedics and Traumatology (SBOT) at the time was contacted and approved of the initiative to be carried out during the event. Additionally, the SBOT press office was contacted to promote the project. Participation in the survey was voluntary, and no incentives were offered to increase the response rate. Both practicing orthopaedic surgeons (n=150) and orthopaedists in training (residents and fellows; n=150) took part in the survey. There was no time limit to take the survey. The participants were informed of the terms of the study (anonymity, purpose, data processing, and identity of the researchers) by means of an informative introductory page at the beginning of the survey and directly by the physicians who carried out the project. The study was written in accordance with the guidelines of the Checklist for Reporting Results of Internet E-Surveys (CHERRIES) [15].

The data were recorded on an Excel spreadsheet (Microsoft Excel, Microsoft® Corp., Redmond, WA, USA) and analysed statistically. Descriptive statistics were reported using frequencies and percentages for categorical data (ordinal and nominal). For the clinical situations of conflict in the workplace, a statistical comparison using the chi-square test was performed, with a 95% confidence interval and a level of significance of 5%. The SAS software (version 9.4, SAS Institute, Cary, NC, USA) was used.

## Results

Participants' profile

A total of 99 women participated in the survey, of whom 66 (66.7%) were practicing orthopaedic surgeons and 33 (33.3%) were orthopaedists in training. Among them, 12 (12.1%) were in training after completing their medical residency (fellowship), and 21 (21.2%) were currently medical residents (p=0.000). Most respondents (57.6%) were either under 30 years old (29.3%) or between 30 and 34 years old (28.3%). Eighty-two (82.8%) participants stated they were Caucasian (p=0.0001). Among the practicing orthopaedic surgeons (n=66), 26 (39.4%) reported working in the private sector, 17 (25.7%) in the public sector (Brazilian Health System), 14 (21.2%) in both the private and academic sectors, three (4.5%) in the military sector, and two (3.0%) solely in the academic sector. All practicing orthopaedic surgeons reported having undergone some fellowship after completing their medical residency. A relatively large number of practicing orthopaedists and those in training after completing their residency opted for paediatric orthopaedics (18, 24.3%) or hand surgery (13, 17.6%) through fellowship programmes. There was no statistically significant difference between the groups (p>0.05). Table 1 presents the profiles of the participants.

**Table 1:** Participants’ profile.

Variables (number of respondents)	Responses	Frequencies and percentages
Stage of training (n=99)	Practicing orthopaedists	66 (66.7%)
	Orthopaedists in training	33 (33.3%)
	Currently in medical residency	21 (21.2%)
	Currently in fellowship or R4	12 (12.1%)
Age group (in years) (n=99)	Under 30	29 (29.3%)
	30 to 34	28 (28.3%)
	35 to 39	16 (16.2%)
	40 to 44	12 (12.1%)
	45 to 50	10 (10.1%)
	51 to 60	4 (4.0%)
Reported ethnicity (n=99)	White	82 (82.8%)
	Multiracial	14 (14.1%)
	Black	2 (2.0%)
	Prefer not to disclose	1 (1.0%)
Work sector (n=66)	Private	26 (39.4%)
	Private and academic	14 (21.2%)
	Public	17 (25.7%)
	Academic (University)	2 (3.0%)
	Military	3 (4.5%)
	Other	4 (6.1%)
Years of practice (n=66)	<5	19 (28.8%)
	6 –10	16 (24.2%)
	11–15	15 (22.7%)
	16–20 >21	7 (10.6%) 9 (13.6%)
Area of interest for fellowship or R4 (n=74)	Reconstruction / External Fixator	2 (2.7%)
	Orthopaedic Oncology	2 (2.7%)
	Foot and Ankle	7 (9.5%)
	Knee	4 (4.5%)
	Hip	4 (5.4%)
	Hand	13 (17.6%)
	Shoulder and Elbow	5 (6.8%)
	Paediatric Orthopaedics	18 (24.3%)
	Spine	4 (5.4%)
	Sports Medicine	7 (9.5%)
	Orthopaedic Trauma	5 (6.8%)
	Pain	3 (4.1%)

Academic and associative experiences

Most participants reported never having a scientific publication (35; 35.4%), and 29 (29.3%) reported publishing only one scientific paper. Only 11 (11.1%) participants reported having five or more publications since the beginning of their practice in the specialty. Most practicing orthopaedists had published between one and five papers, while among those in training, the majority had no publications (p=0.003). Of the 99 respondents, 29 (29.3%) said they were not part of any medical associations related to the specialty. Of the 78 practicing orthopaedists and those in training after completing their residency, 64 (82%) responded that they were full members of SBOT. Of the 70 respondents who reported being members of at least one medical association related to the specialty, the specialty associations most frequently cited were SBOP (Brazilian Society of Paediatric Orthopaedics) and SBCM (Brazilian Society of Hand Surgery), with 14 (20.0%) and 11 (15.7%) reporting these organizations, respectively. Among those who reported being members of at least one medical association related to the specialty (excluding SBOT), various respondents were members of at least two other associations. There was no statistically significant difference between the other variables and groups (p>0.05). Respondents’ number of publications and engagement with academic associations are listed in Table 2.

**Table 2:** Number of publications and academic association engagement. ASAMI: Association for the Study and Application of Ilizarov Methods; ABTPe: Brazilian Association of Medicine and Surgery of the Ankle and Foot; SBCJ: Brazilian Society of Knee Surgery; SBQ: Brazilian Hip Society; SCBM: Brazilian Society of Hand Surgery; SCCOC: Brazilian Society of Shoulder and Elbow Surgery; SBOP: Brazilian Society of Paediatric Orthopaedics; SBC: Brazilian Spine Society; SBRATE: Brazilian Society of Sports Arthroscopy and Traumatology; SBTO: Brazilian Orthopaedic Trauma Association; AMOB: Association of Orthopaedic Women in Brazil; ASPN: American Society for Peripheral Nerve; POSNA: Paediatric Orthopaedic Society of North America; SBUS: Brazilian Society of Ultrasound.

Variable	Frequency and percentage	
Number of scientific studies published (n=99)	None	35 (35.4%)
	One	29 (29.3%)
	Two	11 (11.1%)
	Three	10 (10.1%)
	Four	3 (3.0%)
	Five or more	11 (11.1%)
Full member of the SBOT (n=78)	Yes	64 (82.0%)
	No	12 (18.0%)
Medical associations and committees (n=70)	ASAMI	4 (5.7%)
	ABTPe	3 (4.3%)
	SBCJ	2 (2.8%)
	SBQ	0 (0%)
	SBCM	11 (15.7%)
	SBCOC	2 (2.8%)
	SBOP	14 (20.0%)
	SBC	1 (1.4%)
	SBRATE	5 (7.1%)
	SBTO	4 (5.7%)
	Pain Committee	1 (1.4%)
	AMOB	3 (4.3%)
	ASPN	1 (1.4%)
	POSNA	1 (1.4%)
	SBUS	1 (1.4%)

Experience of conflict in the work environment

When asked if they had been referred to as 'bossy, overly assertive, pushy, overly demanding, or difficult to deal with,' a majority responded yes (7, 70.7%). Among those who responded yes, 46 (65.7%) agreed that these comments were more frequently towards orthopaedists who were women and men; among those who responded no, the majority (8, 27.6%) disagreed that such comments were directed selectively. In terms of level of training, most practicing professionals responded no to the question, whereas a majority of orthopaedists in training responded yes (p=0.020). Regarding the scenario occurring more frequently with orthopaedists from ethnic minorities than with white orthopaedists, 26 (37.1%) of the respondents who answered yes fully agreed with that statement, while 13 (44.8%) of those who responded no disagreed with it. As with the previous question, a majority of practicing orthopaedic surgeons disagreed with the statement, and a minority of orthopaedists in training disagreed with the statement (p=0.025).

When questioned whether they had felt disregarded as a surgeon by a patient when recommending a procedure, 76 (76.8%) responded yes and 23 (23.2%) answered no. When queried about this scenario occurring more frequently with orthopaedists who were women relative to those who were men, among those who responded yes, 52 (68.4%) fully agreed with the statement, while among those who answered no, six (26.1%) fully agreed with the statement. When asked if this scenario occurred more frequently with orthopaedists from ethnic minorities than with white orthopaedists, among those who responded affirmatively, 30 (39.5%) fully agreed with the statement. When inquired if they had achieved their career goals according to their qualifications, 83 (83.8%) responded yes, 15 (15.2%) responded no, and one (1.0%) participant abstained from answering. When asked if this scenario occurred more frequently with women orthopaedists than with orthopaedists who were men, among those who answered no to achieving their career goals, 15 (86.7%) agreed or fully agreed with the statement. A similar situation arose when asked if this scenario occurred more frequently with orthopaedists from ethnic minorities than with Caucasian orthopaedists; among women orthopaedists who responded negatively to achieving their career goals, nine (61.0%) agreed or fully agreed with the statement.

Regarding work schedules, 20 (20.2%) respondents stated that their work hours were not equitably distributed between orthopaedic surgeons who were women and men. Of those respondents, 19 (95.0%) agreed or fully agreed that this scenario occurred more frequently with women orthopaedists than with orthopaedists who were men, and 12 (60.0%) agreed or fully agreed that this issue is more prevalent among orthopaedists from ethnic minorities than among Caucasian orthopaedists. Regarding recognition for their contributions to the department, service, or clinical unit in which they work, 36 (36.4%) respondents reported not feeling recognized, with 30 (83.3%) agreeing or fully agreeing with this scenario. Among the 36 participants who expressed not feeling recognized in their professional environment, 25 (69.5%) agreed or fully agreed that the situation occurs more frequently with orthopaedists from ethnic minorities than with Caucasian orthopaedists. When asked if there were differences in reporting of similar behaviour being reported or charged between orthopaedic surgeons who were women and men, a majority (75, 75.8%) responded no. Among the orthopaedists who responded yes, 19 (79.2%) fully agreed that this scenario occurs more frequently with female orthopaedists than with male orthopaedists. However, respondents did not have a bearing on whether this scenario occurred more frequently with ethnic minorities and Caucasian orthopaedists (15 [62.5%] participants felt neutral about the existence of bias). Finally, when asked whether there was a difference between orthopaedists who were men and women being reported by patients for poor outcomes, 14 (14.1%) responded yes. Most professionals who reported poor outcomes were orthopaedists in training (p=0.045). There was no statistically significant difference between the other variables and groups (p>0.05).

Conflicts in the workplace

**Figure 1:**
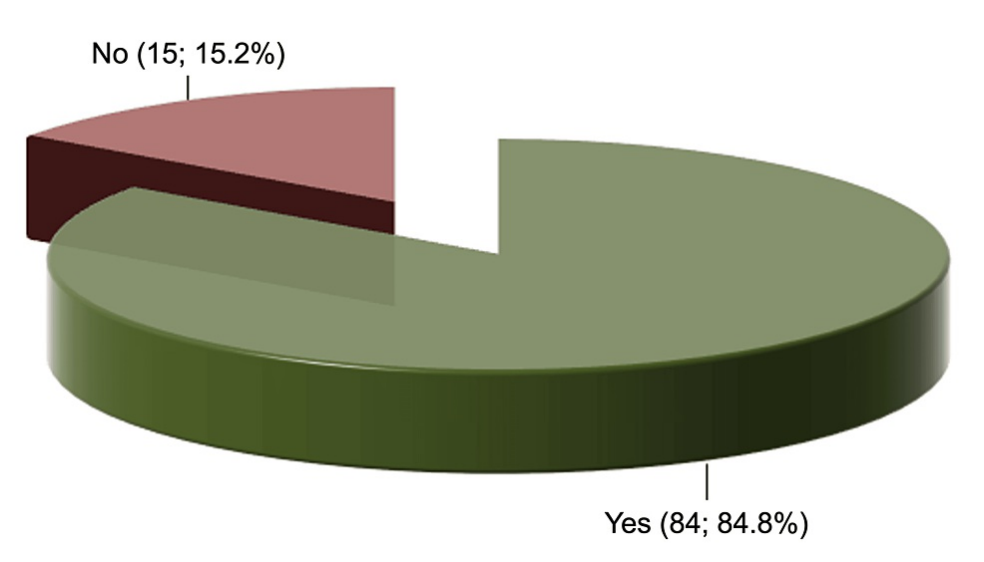
Prejudice from another professional or doubt about women's ability because of gender inequality.

Most orthopaedists reported having experienced some form of professional conflict in their workplace, while only 15 (15.2%) stated they had never experienced any (Figure 1).

**Figure 2:**
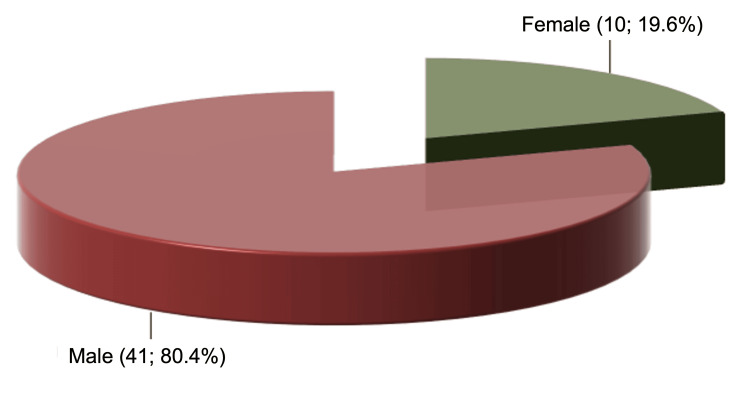
Censure suffered from another person regarding gender.

Among the 84 (84.8%) orthopaedists who reported having experienced professional conflicts, 65 (77.4%) said it was with another orthopaedist, although many mentioned that the situation had occurred with more than one type of professional. Forty-eight (48.5%) participants had never been censured, while five (5.1%) participants had been censured more than 10 times. Among the 51 (51.5%) orthopaedists who reported being censured, the most common source was other orthopaedists (47.1%), and the censuring was predominantly done by professionals who were men (80.4%) and Caucasian (92.2%) (Figure 2).

Three (3.0%) orthopaedists reported receiving disciplinary actions. Among the emotional impacts caused by the censuring received, the most reported ones were anxiety (32.9%), burnout (25.7%), and suicidal thoughts (14.3%). Twenty (28.6%) participants reported experiencing emotional issues that lasted up to six months, while 14 (20.0%) reported that the issues persisted for over a year. Most practicing orthopaedists identified depression as the main emotional impact caused by the censuring received, whereas among orthopaedists in training, anxiety and burnout were the primary emotional impacts (p=0.040). Among the physical impacts caused by the censuring received, the most reported ones were weight gain or loss (29.3%) and insomnia and/or sleep disorders (22.7%). Twelve (17.1%) participants reported experiencing physical issues that lasted up to six months, while 14 (20.0%) reported that the issues persisted for over a year. Out of the 84 orthopaedists who reported experiencing some form of professional conflict, 26 (30.9%) stated that it did not affect their personal relationships with the person involved, while 24 (28.6%) mentioned that their personal relationships were indeed affected, with seven (29.2%) noting changes in workplace communication. Among the orthopaedists who experienced a professional conflict, the majority (83.3%) stated that it did not affect patient care. Twelve (14.3%) orthopaedists reported that their professional trajectory was impacted because of the conflict, with six (7.1%) leaving their jobs for this reason. Most professionals whose professional trajectory was affected were practicing orthopaedists (p=0.012). Regarding career choice, 87 (87.9%) participants stated that they would choose the same profession again, and 81 (81.8%) of them would choose the same specialty. However, most orthopaedists (77.8%) had never been encouraged to participate in personal or professional development programs. Among the suggestions provided to improve gender equity among orthopaedists, the presence of women in leadership positions in orthopaedic services was mentioned by 11 (8.5%) participants. There was no statistically significant difference between the other variables and groups (p>0.05). Table 3 presents the data related to workplace conflicts.

**Table 3:** Conflicts in the workplace.

Professional conflict (n=99)	Yes	84 (84.8%)
	No	15 (15.2%)
Professionals who caused the conflict (n=84)	Another orthopaedist	65 (41.9%)
	Non-orthopaedic physician	44 (28.4%)
	Another healthcare professional (non-physician)	46 (29.7%)
Number of conflicts resulting in censuring (n=99)	0	48 (48.5%)
	1 to 3	35 (35.4%)
	4 to 6	8 (8.1%)
	7 to10	3 (3.0%)
	More than 10	5 (5.1%)
Emotional impacts (n=84)	None	6 (8.6%)
	Depression	4 (5.7%)
	Anxiety	23 (32.9%)
	Suicidal thoughts	10 (14.3%)
	Burnout/reduced job satisfaction	18 (25.7%)
	Panic disorder	1 (1.4%)
	Rage	1 (1.4%)
	Insecurity	1 (1.4%)
	Constant worry	1 (1.4%)
	Fear of work	1 (1.4%)
	Not applicable	2 (2.9%)
	Did not disclose	1 (1.4%)
Physical impacts (n=84)	None	12(16.0%)
	Weight gain or loss	22 (29.3%)
	Insomnia/sleep disorders	17 (22.7%)
	Headaches	8 (10.7%)
	Alopecia	6 (8.0%)
	Acne/skin problems	5 (6.7%)
	Not applicable	3 (4.0%)
	Did not disclose	2 (2.7%)

## Discussion

Gender inequality among medical professionals is a significant issue worldwide [3,4,16]. While both women and men face difficulties and challenges in their professional lives, women continue to play more roles outside of work than their counterparts, who were men in the globally established patriarchal model [17]. These multifaceted roles have been used to rationalize gender gaps in organizational, professional, and academic opportunities, characterized by lower women's representation in various surgical specialties, including orthopaedics. Despite the number of women in medical schools having surpassed that of men for over 25 years [10,16], specialization in orthopaedic surgery by women remains minimal, with men accounting for over 95.0% of all orthopaedic surgeons worldwide [18]. In addition, historically, women physicians have not achieved equal representation in leadership positions, are less likely to reach higher positions regardless of age, experience, or academic achievement, and continue to experience numerous professional conflicts in their work environment [9,16]. In the present study involving 99 Brazilian women orthopaedists both practicing and in training who voluntarily responded to the questionnaire, we observed a low volume of publications, moderate involvement in professional associations, and a high number of conflicts experienced in the work environment. To the best of our knowledge, this is the first study conducted in Brazil with the aim of understanding barriers to gender equity in the orthopaedic surgical specialty. The hope is that issues can be clarified, with steps to mitigate gaps initiated.

As evidenced by the relatively few women in leadership positions in medical specialties, the existing support for women to progress in their careers is insufficient and even more critical for minority groups, where there is an evident paucity of role models and mentors in academic life [16]. In this context, additional efforts are needed to provide women surgeons with more professional opportunities, including increased numbers of sponsorships, academic incentives, publications, citations, and collaborations [19]. While highlighting gender inequalities in research and publishing may seem biassed due to the greater number of men in the field [19], women orthopaedic surgeons publish less and have fewer chances to contribute as guest authors than their counterparts who are men, many of whom have similar professional experiences and positions [20]. In a cross-sectional study from 1987 to 2017, based on six major orthopaedic scientific publications (Journal of Bone and Joint Surgery, Journal of Arthroplasty, Journal of Orthopaedic Trauma, American Journal of Sports Medicine, Journal of Hand Surgery, and Journal of Paediatric Orthopaedics), Brown et al. observed that 1.7% of senior authors and 4.4% of first authors were women [20]. In another cross-sectional study conducted among 713 Canadian academic orthopaedic surgeons, Yue and Khosa revealed that men hold 87.0% of academic positions, and women are less likely to hold leadership positions [21]. Furthermore, these authors observed that Canadian women orthopaedists are underrepresented in terms of numbers, positions, and academic productivity. In another cross-sectional study, Khalifa et al. [22] compared the number of authors per article and the prevalence of women authors in two orthopaedic journals from the Arab world: the Archives of the Egyptian Orthopaedic Journal (EOJ) and the Journal of Musculoskeletal Surgery and Research (JMSR). These authors reviewed all articles published prior to July 2020 and found that the prevalence of women authors in the JMSR was significantly higher than in the EOJ (14.2% versus 0.3%, respectively). In the current study, 35.4% of the participants reported never having published a scientific paper, 29.3% reported having published only one scientific paper, and only 11.1% reported having five or more publications since the beginning of their practice in the specialty. Most practicing orthopaedists had published between one and five papers, while among orthopaedists in training, most did not have any publications (p=0.003). These findings shed light on the need for institutional support for more active participation in scientific research and publication by women physicians, starting early in their education and specialty training. It is widely recognized that being up-to-date with scientific advancements and actively engaging in academic activities are crucial factors for professional advancement in various orthopaedic disciplines [22].

In this context, another interesting finding of the present study was a moderate level of involvement in academic societies among the respondents. Out of the 99 participants, 70.7% reported being members of at least one medical association related to the specialty, with 82% of practicing orthopaedists and those undergoing post-residency training reporting affiliation to the SBOT. Among the most cited subspecialty associations were the SBOP and SBCM. According to data provided by the SBOT for the year 2022, out of 13,159 active members, 835 (6.3%) were women, and among the 1,755 medical residents in the specialty, 383 (21.8%) were women. Other countries in the region show similar numbers of women orthopaedic surgeons, with 6.2% in Chile and slightly higher rates in Mexico (9.2%) and Colombia (9.8%) [8,23], but the lack of gender diversity in orthopaedic surgery is not exclusive to Brazil and other Latin American countries. Recent demographic data published by the International Orthopaedic Diversity Alliance reveal that 12.0% of practicing orthopaedic surgeons are women in Canada, 6.5% in the United States (US), 4.8% in the United Kingdom, and only 0.5% in India and Nepal [23]. Regarding women orthopaedists in training, in the US, women represent 16% of orthopaedic residents and 4% of fellows, while in Canada they represent 13.2% of orthopaedic residents [24]. The relatively low number of women in orthopaedics and their consequent underrepresentation in professional associations perpetuate current gender disparities and represent a barrier to women from holding positions of academic leadership and prominence [25]. According to a study evaluating women’s participation during the annual meetings of the American Academy of Orthopaedic Surgeons (AAOS) in 2009, 2014, and 2019, only 6.8% of the 3,980 oral presentations were conducted by women, with the majority of them being related to non-technical sessions [24]. The parallel lack of representation of women physicians in specialty societies and associations, academic medicine, and leadership positions reveals a direct relationship between the representation of women physicians and their status in medicine [1,8,9]. In a retrospective cohort study, Silvestre et al. analyzed the academic achievements and demographic profiles of the presidents of the AAOS, the American Orthopaedic Association (AOA), and the American Board of Orthopaedic Surgery (ABOS) elected from 1990 to 2020 [25]. Eighty presidents were included, with the majority being men (97%) and only two women holding those positions (one in ABOS in 2012 and one in AAOS in 2019). Additionally, only 4% of the presidents were from racial minorities (3% African Americans and 1% Hispanics). In Brazil, since 1935, when Luiz Manoel Rezende Puech took office as the first president of the SBOT, only one woman orthopaedic surgeon has held that position in the largest and most important orthopaedic medical association in the country [17]. Greater visibility, recognition, and representation can only occur through the implementation of career and leadership development programmes, which, based on projected analysis, can ensure models for promoting identity compatibility and social and professional belonging [16,25].

Achieving equity in academic and professional roles can also help reduce gender conflicts in the specialty. Rodarte et al. evaluated the experiences of North American women orthopaedic surgeons in dealing with workplace conflicts using a questionnaire on which we based the present study [9]. Out of 373 respondents, 72.0% described experiencing some form of work-related conflict self-attributed to their gender, leading to depression, anxiety, and burnout, and 17.0% said they would not choose the same career again if given another professional opportunity. Among orthopaedists in training, the scenario is no different, with a significant risk of friction. Haruno et al. investigated the trends in racial and gender diversity and in friction rates in orthopaedic training programmes from 2001 to 2018 [26]. Among women residents, the rates of overall and unintentional friction were 6.0% and 2.1%, respectively, while among residents who were men, they were 2.8% and 1.0%, respectively. Among residents from ethnic minorities, the rates of overall and unintentional friction were 6.2% and 3.1%, respectively, compared to 2.7% and 0.8%, respectively, for their colleagues of white etnicity [26]. In our study, 84.8% of women orthopaedists reported having experienced some form of professional conflict, with the majority indicating that the conflict occurred with another orthopaedist, and 70.7% said they had been referred to as 'bossy, overly assertive, pushy, overly demanding, or difficult to deal with'. Compared to practicing women orthopaedic surgeons, most women orthopaedists in training agreed with the statement that this scenario of professional conflicts happened more often with women orthopaedists than with orthopaedists who were men (p=0.020), and more often with orthopaedists from ethnic minorities than with Caucasian orthopaedists (p=0.025). Other studies have shown high awareness among orthopaedists, both practicing and in training, of harassment, assault, and gender and ethnic discrimination in the workplace [27,28].

Even though people process discrimination differently, workplace conflicts have been observed to have negative repercussions for women physicians, often leading to long-term physical and psychological distress. In their study, Rodarte et al. [9] reported that 8% of the participants described being forced to resign or leave their previous jobs due to workplace conflicts, reinforcing the observation that not all women orthopaedic surgeons can tolerate the conflicts they experience on a daily basis. In the current study, half of the sampled orthopaedists reported experiencing censure, primarily from their Caucasian colleagues. Anxiety and burnout were the most frequently reported emotional impacts among orthopaedists in training, while depression was the most common emotional impact among practicing orthopaedic surgeons. As for physical impacts, the most common were body weight fluctuations and sleep disorders. Twelve orthopaedists, most of them in practice (p=0.012), stated that their professional trajectory was negatively affected due to some form of professional conflict, with six leaving their jobs for that reason. Nonetheless, 87.9% of the participants said they would choose the same profession again, and 81.8% of them would choose the same specialty. Our finding is quite similar to the one presented in the most recent Physician Compensation Report of 2023, conducted with American physicians, in which 96.0% of orthopaedists said they would choose the same specialty again [29]. This finding is quite different from the study by Rodarte et al. [9], also conducted with North-American women orthopaedists, where one in five participants reported that they would not choose the same profession or specialty again. Being a woman may be the main explanation for the significant difference observed between the two American studies on the same subject.

In this context, one of the most notable findings of the present study is the observation that strategies, actions, and opportunities for inclusion are needed for practicing women physicians and those in training, as well as undergraduate students, to enable more active participation in the specialty. In Brazil, the SBOT and other subspecialty associations have been stimulating and promoting gender equity and equality, while other orthopaedic associations in different parts of the world have also been making similar efforts to gradually reduce the barriers that hinder gender diversity [1,9,18,23,24,30]. Sachdeva et al. [18] mention the existence of national outreach programmes during undergraduate education that provide early exposure to orthopaedics for women and other underrepresented minorities in the USA. The presence of a larger number of women professors and mentors in undergraduate and medical residency programmes can signal to potential female candidates that surgical specialty programmes, such as orthopaedics, are committed to creating an inclusive space for all candidates. Furthermore, all medical school curricula should include orthopaedic surgery as a mandatory component in order to provide more opportunities for early exposure to the specialty for female students [18]. Finally, the implementation of more substantial parental rights and progressive social policies can encourage greater participation by women in orthopaedics [23]. Other strengths of our study include the use of a questionnaire containing direct, simple, and comprehensive questions, which makes it implementable in other settings, requiring only a few adjustments for the region or country in which it is used. Additionally, this study is the first initiative of its kind among Brazilian orthopaedic surgeons, and our findings.

Although our study yielded consistent findings, it had several limitations. The first and major limitation of the study is the relatively low number of female respondents, despite the fact that there are 835 practicing women orthopaedists and 383 in training in Brazil, as well as an underrepresentation of ethnic minorities. Other authors have also highlighted the small number of responses and the low representation of non-white ethnicities as limitations in their studies [9]. Other Brazilian authors showed a low response rate among Brazilian women doctors in similar studies, always involving issues of relevance and gender inclusion [31,32]. This is curious, as women are generally more likely to contribute to and respond to surveys than men. In our study, the response rate was only 30%, meaning the study suffered from a 70% non-response bias. Even though we know that the response rate is extremely important when it comes to representation and specifically gender diversity, it is a topic of growing discussion in orthopaedics. The lack of time, the discredit in short- and medium-term changes, and the communication error between researchers and respondents must be recognized as potential causes for the reduced number of responses. Research has also shown that, in general, surveys targeting physicians tend to have lower response rates [33]. Nevertheless, we believe that the current study supports the idea that Brazilian women orthopaedic surgeons would benefit from active measures to improve equity, inclusion, and innovation in their specialty organizations. A second limitation is the lack of validation of the survey used. Some bias could be generated when comparing our responses with the same questionnaire applied to populations with different languages and/or cultures. Future studies can be carried out after validating the questionnaire for Brazilian Portuguese, with the aim of increasing the number of respondents.

## Conclusions

Our study showed that women orthopaedic surgeons in training in Brazil have a lower number of publications and a moderate level of involvement in academic society activity. In addition, orthopaedic surgeons in training experience a statistically significant higher number of conflicts in the workplace. The comments from the questionnaires highlighted the physical and psychological consequences arising from these situations of professional conflict. In general, conflicts occur more frequently with Caucasian orthopaedists who are men, indicating a role for women’s feelings of inequality in the workplace that ultimately reduces the level of satisfaction and may contribute to disinterest and abandonment of the specialty. This was more pronounced in the youngest orthopaedist populations and may reflect the persistence of inequalities between genders, despite the numerous behavioural and educational changes that have been implemented in societies around the world. The current study’s findings support recent evidence that there is an implicit and often overlooked bias against the participation of women and ethnic minorities in the orthopaedic community, an issue that calls for increased efforts to achieve inclusion and diversity.
